# Unilateral zebrafish corneal injury induces bilateral cell plasticity supporting wound closure

**DOI:** 10.1038/s41598-021-04086-x

**Published:** 2022-01-07

**Authors:** Kaisa Ikkala, Vassilis Stratoulias, Frederic Michon

**Affiliations:** 1 Institute of Biotechnology, HiLIFE, University of Helsinki, Helsinki, Finland; 2 Neuroscience Center, HiLIFE, University of Helsinki, Helsinki, Finland; 3 Institute for Neurosciences of Montpellier, Univ Montpellier, INSERM, Montpellier, France

**Keywords:** Regeneration, Cell signalling, Mechanisms of disease

## Abstract

The cornea, transparent and outermost structure of camera-type eyes, is prone to environmental challenges, but has remarkable wound healing capabilities which enables to preserve vision. The manner in which cell plasticity impacts wound healing remains to be determined. In this study, we report rapid wound closure after zebrafish corneal epithelium abrasion. Furthermore, by investigating the cellular and molecular events taking place during corneal epithelial closure, we show the induction of a bilateral response to a unilateral wound. Our transcriptomic results, together with our TGF-beta receptor inhibition experiments, demonstrate conclusively the crucial role of TGF-beta signaling in corneal wound healing. Finally, our results on *Pax6* expression and bilateral wound healing, demonstrate the decisive impact of epithelial cell plasticity on the pace of healing. Altogether, our study describes terminally differentiated cell competencies in the healing of an injured cornea. These findings will enhance the translation of research on cell plasticity to organ regeneration.

## Introduction

The camera-type eye structure is generally conserved across species. The main innovation of this eye type was the generation of the lens and cornea. Both structures are transparent and of ectodermal origin. While the refractive lens serves only to focus light precisely onto the retina^1^, the cornea is a thin structure serving several roles. Among those roles, the most important are the refraction of light and the physical protection of the eye.

In terrestrial animals, the tear film is one of the sources of hydration and nutrients for the epithelium^2^. When the tear film is chronically defective, corneal surface is harmed, which can lead to progressive vision loss^3^. For obvious reasons, in an aquatic environment, the tear film is lacking, and the source of nutrients for corneal epithelium is currently unknown.

Being the most external tissue of the eye, the cornea must withstand direct challenges from the external environment. Corneal abrasions are very common, and lead to a remarkable influx of patients in emergency room^4^. These abrasions are often caused by small foreign objects, such as dust, sand or other blowing debris, that can induce a scratch on the corneal epithelium. The resulting corneal injury causes physical and functional discomfort. The subsequent edema provokes photophobia and decreased visual acuity^5^. In case of a deeply embedded foreign object, corneal irregularities can occur, resulting in significant visual disruption.

Corneal epithelial wound healing encompasses several processes, including cell migration, proliferation, adhesion and differentiation^6,7^, which are regulated via various molecular cues (such as growth factors, cytokines, and neuropeptides)^8^. We have previously shown that murine corneal wound closure is based on cell rearrangements^9^. We have shown as well that tear composition is quickly modified following corneal injury, to support corneal healing^10^. These changes in the corneal microenvironment, are crucial for proper wound closure, by supplying a new combination of factors via the tears when necessary.

**Figure 1:**
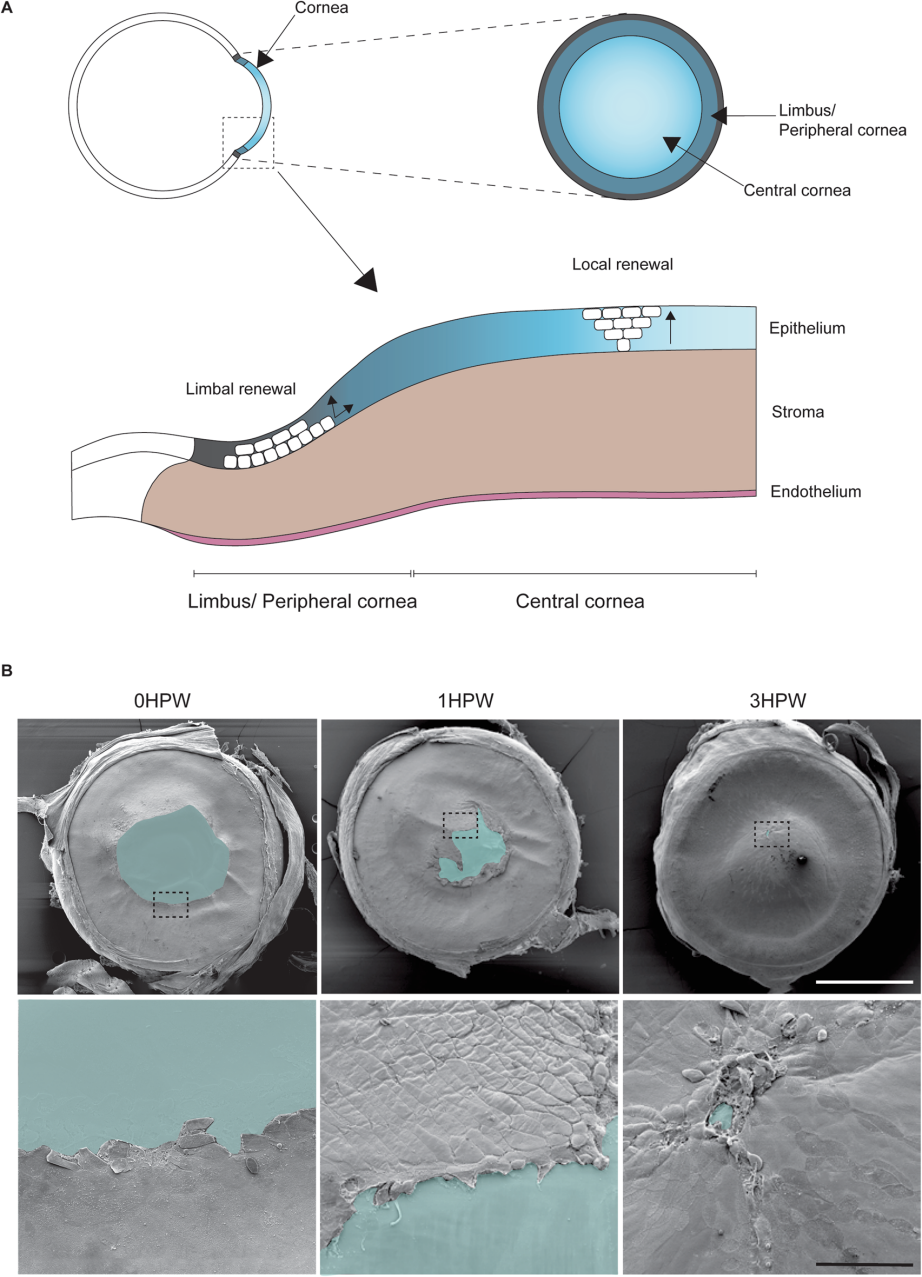
Overview of the cornea and the early steps of corneal wound healing in zebrafish. (**A**) Schematic presentation of the corneal regions, cell compartments, and epithelial renewal. (**B**) Scanning electron microscopy images display clearly the corneal epithelial wound at 0, 1, and 3 h after abrasion. Upper panel: overview of the eyes, blue color marks the wounded area. Lower panel: close up on wound border corresponding to the dashed line boxes from upper row. Three hours after abrasion, the epithelial wound is about to be completely closed. Scale bars: 500 µm upper panel, 50 µm lower panel. HPW: hours post-wound.

Fish cornea are anatomically similar to mammalian ones (Fig. 1), distinguished only by minor adaptations whose purpose is to withstand pressure, such as is seen in tuna^11^. The most posterior cell layer is the endothelium, which lines the cornea and regulates the exchange of liquid with the anterior chamber of the eye. Next, the stroma is situated between the epithelium and endothelium. This part consists of highly organized collagen fibers, and contains only a few cells. The structural organization of these fibers is necessary for corneal transparency. Finally, the cornea’s most external tissue is the corneal epithelium, which throughout its life undergoes a process of continuous renewal^12^. In mice, the central cornea is a disc covering about 60% of the total corneal surface in mouse^9^. This is composed of 3 to 6 cell layers. The peripheral/limbal region of the murine corneal epithelium takes the form of a ring around the central cornea. This region contains the corneal epithelial stem cells, and is known to have a more active proliferation rate than the central region^9,13,14^. These stem cells are responsible for the global renewal of the corneal epithelium. Cells produced by stem cells differentiate during their transit towards the corneal center, through a global centripetal movement^15^. Corneal injury has been shown to increase limbal/peripheral proliferation and centripetal movement, to support corneal repair^16,17^. Some reports, however, have indicated potential in the central corneal cells to resolve epithelial injuries^18^ and to replenish the stem cell pool after limbal injury^19^. More detailed analysis on corneal epithelial cell populations^20,21^, and the impact of the magnitude of the injury^22^, are providing new insigths in the mechanisms of corneal wound healing. An elegant study demonstrated that the dynamics of epithelial renewal are similar in zebrafish and mammalian cornea^23^. It is thus of great interest to investigate the corneal wound healing in this aquatic model organism.

In this study, we characterized the early stages of corneal epithelial wound healing in zebrafish, with an emphasis on cell morphology and dynamics. Our findings indicated that in homeostasis, there was a clear distinction between the central and peripheral cornea as to the cell area and microridge abundancy, reflecting the renewal dynamics of the corneal epithelium. As shown in other epithelial wound healing models, cellular rearrangements formed the initial driving force for wound closure. In zebrafish cornea, however, the response was remarkably fast. A transcriptomic analysis of the repairing cornea showed a transcription modulation of TGF-beta signaling pathway elements, and a downregulation of *Pax6* mRNA and protein, suggesting dedifferentiation to be a key mechanism in the healing process. Furthermore, we observed a similar response on the contralateral unharmed cornea which revealed itself to lead to a faster wound closure after abrasion. These results broaden the understanding of corneal wound healing across species, and contribute to the identification of potential therapeutic targets for treating corneal defects.

## Results

### Epithelial wound closes rapidly after corneal abrasion

To analyze the corneal wound process in zebrafish, we adapted the mechanical abrasion methodology we had previously used in rodents^9^. We used an ophthalmological burr to remove the central corneal epithelium. We have previously shown that in mice the mechanical abrasion leads to a full removal of epithelial cells at the wound site, with no obvious impact on the underlying stroma (Fig. S1). To gain insights into the epithelial wound healing timeframe, we used Scanning Electron Microscopy (SEM) (Fig. 1B). Immediately after mechanical wounding, the resulting abrasion typically covered most of central cornea (over half of the corneal diameter, with an average diameter of 0.77 mm for the wound, and 1.5 mm for the cornea). In as little as 1 h (hr) post-wound (1HPW), epithelial cells already covered a part of the abraded area. The wound edges moved rapidly towards each other. Within 3 h (3HPW), the wound was either completely sealed or close to being completely sealed (Fig. 1B). This time frame was confirmed by histological sections (Fig. S1). Using SEM, we here convincingly demonstrated that corneal closure in zebrafish is completed within 3HPW. To explain this rapid reaction, we then investigated the mechanisms involved during corneal wound closure.

### Cell proliferation is induced after abrasion, but is not required for wound closure

**Figure 2:**
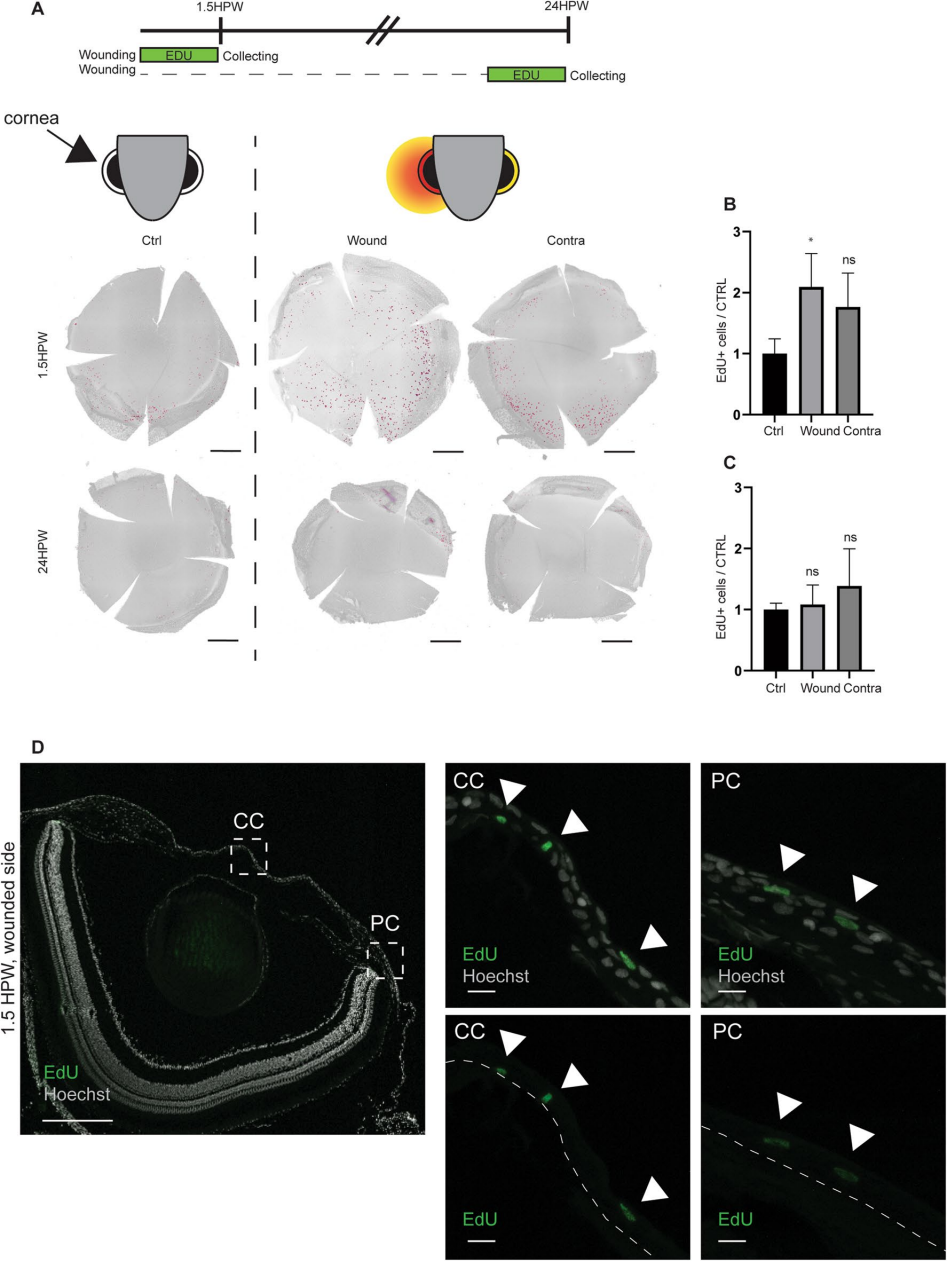
Induction of cell proliferation after epithelial abrasion. (**A**) EdU incorporation is performed 1.5 h prior to sample collection, which happens 1.5 or 24HPW. The EdU labelling on whole cornea on wounded side and contralateral side shows proliferating cells mainly in the periphery. (**B**,**C**) The quantification of EdU-positive cells at 1.5HPW (**B**), or at 24HPW (**C**), demonstrates a transitory increase of cell proliferation at 1.5HPW on the wounded side. n = 3 per group. (**D**) EdU signal with nuclear counterstaining on formalin-fixed, paraffin-embedded section (5 µm) shows the presence of EdU+ cells in all epithelial layers. Left: Overview of an EdU-labeled, wounded cornea at 1.5HPW. Right: Close-ups of the areas indicated with dashed line on central (CC) and peripheral (PC) cornea. Upper row: EdU+ cells (green, arrowheads) with Hoechst-staining, lower row: Edu+ cells only. Quantification data represent mean + stdev, *P < 0.05, Kruskal–Wallis test followed by Dunn’s test for multiple comparisons. Scale bars: 300 µm in A, 200 and 10 µm in D. HPW: hours post-wound.

**Figure 3:**
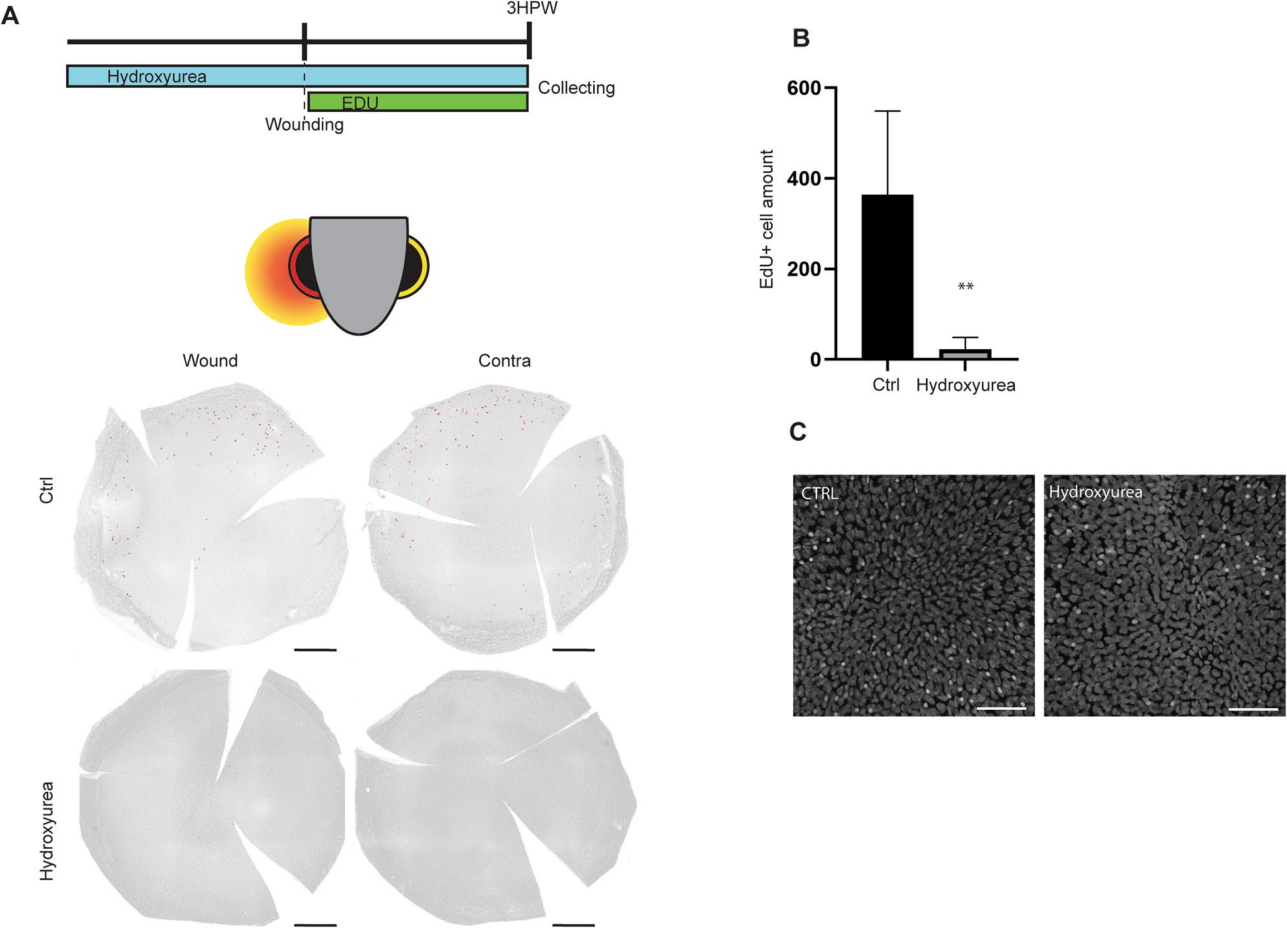
Impact of hydroxyurea on proliferation and wound closure. (**A**) While Hydroxyurea treatment is performed during 6 h (3 h pre- and 3 h post-abrasion), EdU incorporation is performed during the 3 h post-abrasion. Whole mount staining of EdU+ cells reveals the arrest of EdU incorporation due to hydroxyurea treatment. (**B**) The quantification of the EdU+ cells from (**A**) confirms the significant arrest of EdU incorporation under hydroxyurea treatment, n = 6 per group. (**C**) Hoechst staining on wound site in control versus hydroxyurea-treated animal demonstrated a complete wound closure, and the absence of potentially defected cells. Quantification data represent mean + stdev, **P < 0.01, , Mann–Whitney test (exact *p* value, two-tailed) for comparing two groups. Scale bars: 300 µm in A, 50 µm in C. HPW: hours post-wound.

To understand the mechanisms which occur during the corneal wound closure, we investigated the cellular events driving epithelial closure. We had previously shown that in mice cell proliferation is not involved in the closing of wounds after abrasion^9^. To assess the requirement of epithelial cell proliferation in zebrafish during corneal wound closure, we used EdU (5-ethynyl-2′-deoxyuridine) to label DNA synthesis^24^. We added EdU to the tank water for 1.5 h (Fig. 2), either immediately following corneal abrasion, or at 22.5HPW, by which time we hypothesize that the corneal epithelium was healed, based on our previous work on murine cornea^9^. At 1.5HPW, the abraded cornea displayed twice as many proliferating cells as the cornea of a zebrafish that had been handled in the same way but without the abrasion (Fig. 2A–B). This increased proliferation was found mainly in the peripheral cornea, and more rarely in the central cornea (Fig. 2A,D). Interestingly, the contralateral cornea reacted to physical abrasion. While a higher number of proliferating cells was found on the contralateral side, this observation was not deemed to be significant, due to a large variability in the response. At 24HPW, the proliferation has returned was back to the control level, reflecting a healing phase shorter than 24 h, and confirming our hypothesis regarding the wound healing period (Fig. 2C) . As the proliferation appeared to be important for wound healing during the early steps of wound closure, we blocked proliferation by adding hydroxyurea to the water tank, as reported earlier^25^. We validated this approach by looking for EdU+ cells after abrasion (Fig. 3). Hydroxyurea was a potent proliferation inhibitor. Close to no proliferating cells were found on corneas after treatment (Fig. 3A,B). An unexpected finding was that, while proliferation was induced by abrasion (Fig. 2B), the inhibition of proliferation by hydroxyurea had no effect on wound closure at 3HPW (Fig. 3C).

### Changes in cell morphology reflect rearrangements required during closure

**Figure 4:**
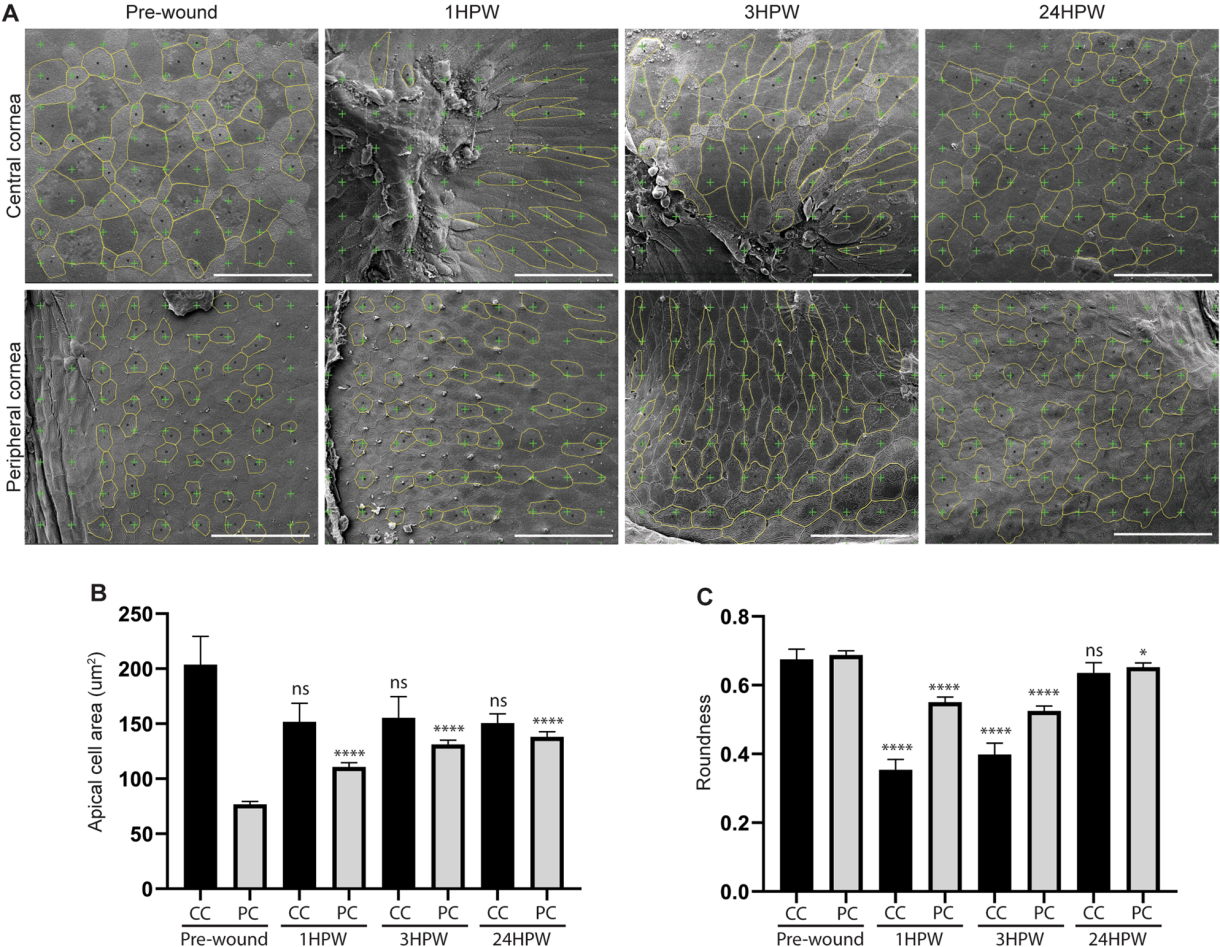
Changes in apical cell area and roundness on wounded cornea. (**A**) Representative SEM images showing cell shape descriptor analysis before wounding, and 1-, 3-, and 24HPW on central and peripheral regions. (**B**) The quantification of apical cell area shows a significant increase of apical cell area on peripheral cornea after wounding, and during 24 h. Cells from 3 eyes were pooled for analysis. (**C**) The quantification of roundness shows a significan decrease of cell roundness in peripheral and central cornea at 1 and 3 HPW. At 24 HPW, the decrease was significant only in the peripheral cornea. Cells from 3 eyes were pooled for analysis. Data represent mean + 95% confidence interval, P** < 0.01, P*** < 0.001, P*** < 0.0001, Kruskal–Wallis test followed by Dunn’s test for multiple comparisons. For both B and C, n = 106, 85, 84, and 95 on CC, and 442, 461, 510, and 492 on PC, for pre-wound, 1HPW, 3HPW, and 24HPW, respectively. CC: central cornea, PC: peripheral cornea. Scale bars: 50 µm. HPW: hours post-wound.

As the rapid wound closure could not be attributed to cell proliferation, we sought to identify another cellular effect which might be responsible for closing the abraded area. We had previously identified cell rearrangements as the driving force behind corneal wound closure, subsequent to epithelial abrasion in mice^9^. Therefore, we monitored cell shape using two parameters, apical cell area and cell roundness. Superficial cells are bigger in the central cornea and smaller on peripheral cornea, where most of the proliferation occurs; the apical cell area should therefore reflect changes in cellular behaviour. Moreover, as the epithelial cell shape is well defined in cohesive epithelia, the cell roundness parameter should mirror population movements. Cells were computationally analyzed for both parameters, on peripheral and central cornea, before and after abrasion (Fig. 4A). Amongst our notable findings was the fact that, while peripheral cornea cells enlarged their apical area rapidly during wound closure (Fig. 4B), their shape changed slightly, albeit after a delay (Fig. 4C). Inversely, central corneal cells, which are closer to the wound site, did not change in size (Fig. 4B), but elongated their axis toward the wound site, and therefore lost their roundness (Fig. 4C). Peripheral corneal cell size continued to be affected at 24HPW, reflecting a long-term effect and longer healing than previously expected. These shape parameters reflected important modificationswhich contributed to closing the wound.

**Figure 5:**
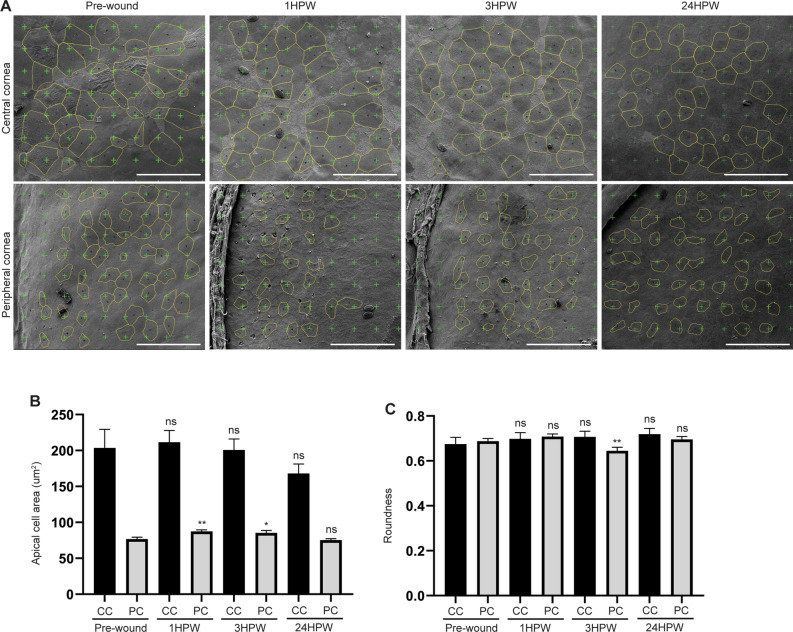
Changes in apical cell area and roundness on contralateral cornea. (**A**) Representative SEM images showing cell shape descriptor analysis before wounding, and 1-, 3-, and 24HPW on central and peripheral regions. (**B**) The quantification of apical cell area shows a slight increase of this parameter at 1 and 3 HPW on the contralateral eye, in peripheral cornea. Cells from 3 eyes were pooled for analysis. (**C**) The quantification of roundness shows a slight decrease of roundness at 3 HPW in peripheral cornea of the contralateral eye. Cells from 3 eyes were pooled for analysis. Data represent mean + 95% confidence interval, P** < 0.01, P*** < 0.001, P**** < 0.0001, Kruskal–Wallis test followed by Dunn’s test for multiple comparisons. For both B and C, n = 106, 109, 129, and 72 on CC, and 442, 481, 362, and 364 on PC, for pre-wound, 1HPW, 3HPW, and 24HPW, respectively. CC: central cornea, PC: peripheral cornea, HPW: hours post-wound. Scale bars: 50 µm. HPW: hours post-wound.

As we identified a slight effect of abrasion on contralateral corneal cell proliferation (Fig. 2B), we analyzed the same shape parameters on contralateral cornea during wound closure (Fig. 5A). Unexpectedly, despite the tight cohesion of epithelial corneal cells, peripheral corneal cells increased their apical area (Fig. 5B), and slightly elongated their axis at 3HPW (Fig. 5C). During the same period, central corneal epithelial cells did not display any significant shape modification. By 24HPW, cell shape parameters had returned to normal.

### Apical microridges are involved in wound repair

**Figure 6:**
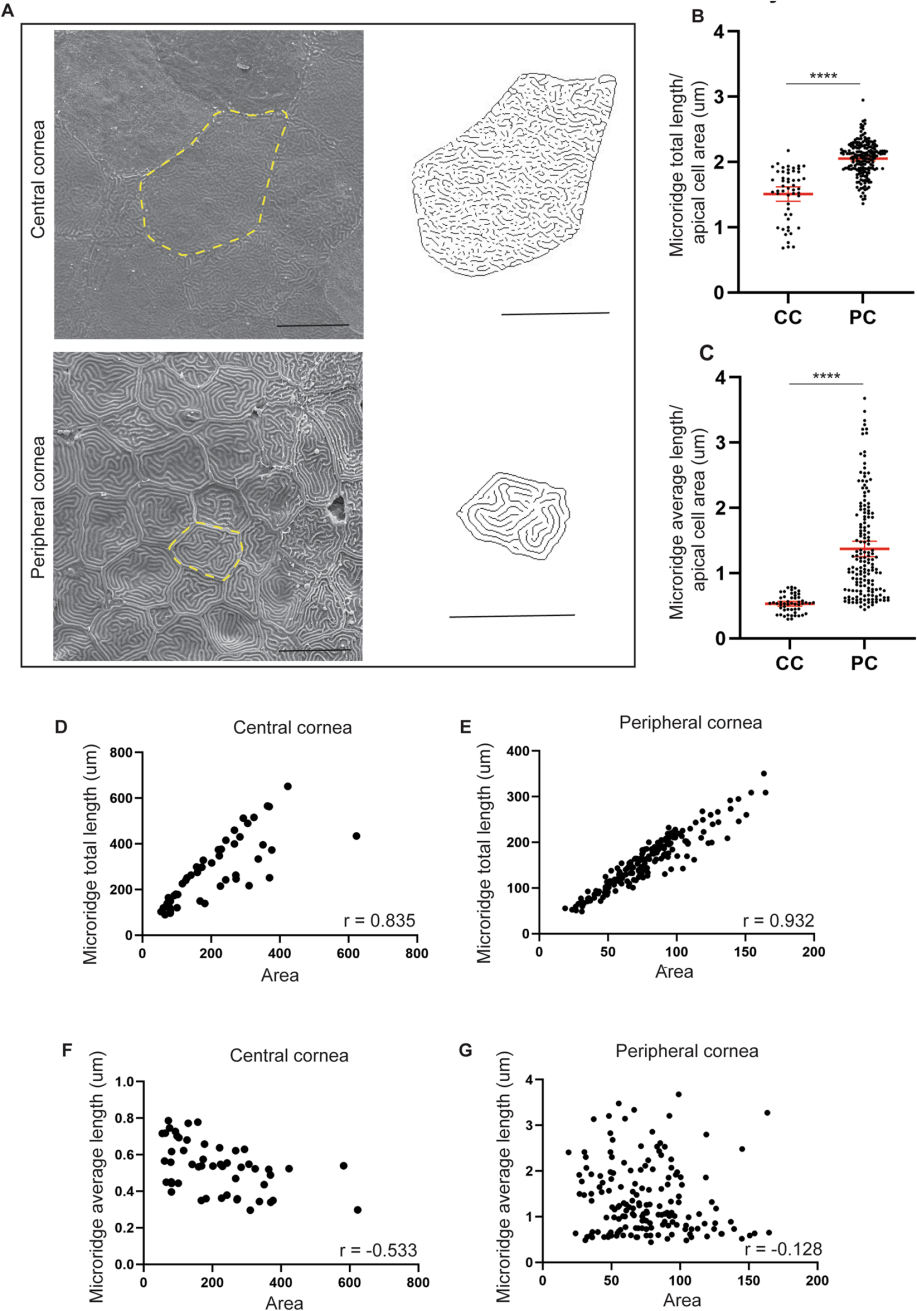
Measures of microridges on zebrafish cornea. (**A**) Representative SEM images on central and peripheral cornea on the left. On the right, the digitally treated images used to measure the parameters of microridges. (**B**,**C**) Average microridge amount (**B**), and length (**C**), per apical cell area on central and peripheral cornea (n = 52 for CC in (**B**,**C**), n = 192 for PC in (**B**), and n = 162 for **PC** in (**C**) after removal of outliers). These parameters are systematically higher in peripheral cornea compared to central cornea. Data represent mean + 95% confidence interval, P**** < 0.0001, Man-Whitney test, two-tailed, with exact *p* value. (**D**–**G**). Correlation of microridge amount or average microridge length, and apical cell area, on central and peripheral cornea (n = 50 in D, 51 in E, 192 in F, and 162 in G, after removal of outliers). While the total length displays a correlation with cell apical area, the average length of microridges offers no correlation with cell apical area. Spearman correlation, two-tailed, with approximate *p* value, P**** < 0.0001. Cells from 3 eyes were pooled for every analyses. Scale bars: 10 µm.

Because cell shape was affected during corneal wound closure, we investigated another element of zebrafish epithelial cell morphology, the microridges. Like other aquatic animals, zebrafish are covered with mucus, decreasing the water friction on their body. This viscous layer also offers protection from the external environment. To maintain this mucus, superficial cells possess apical protrusions, which increase their surface area, and consequently increase their contact with the mucus layer^26^. These actin-based protrusions are subjected to a well-controlled turnover, which increases during the wound-healing process^27^. Superficial corneal epithelial cells have such protrusions^28^. In investigating the zebrafish microridges, we focused on two parameters; their total length, and their average length on each cell’s apical surface (Fig. 6). The first parameter describes how the cell surface is increased by the microridges, while the second parameter indicates the microridges’ level of stability. We discovered that peripheral corneal cells presented well-defined and stabilized microridges compared to central corneal cells (Fig. 6A). To rule out the possibility this phenomenon could be explained by cell size, we normalized the data to cell area. For both parameters (i.e. microridges’ total length (Fig. 6B) and average length (Fig. 6C)), a significant difference was found between central and peripheral corneal cells. We analyzed a possible correlation between these parameters and cell area (Fig. 6D–G). Interestingly, we found that while microridge total length was largely dependent on cell size, in central and peripheral cells (Fig. 6D–E), their average length was not (Fig. 6F–G). This indicates that microridge stability is specific to cell type (peripheral or central) rather than cell size.

**Figure 7:**
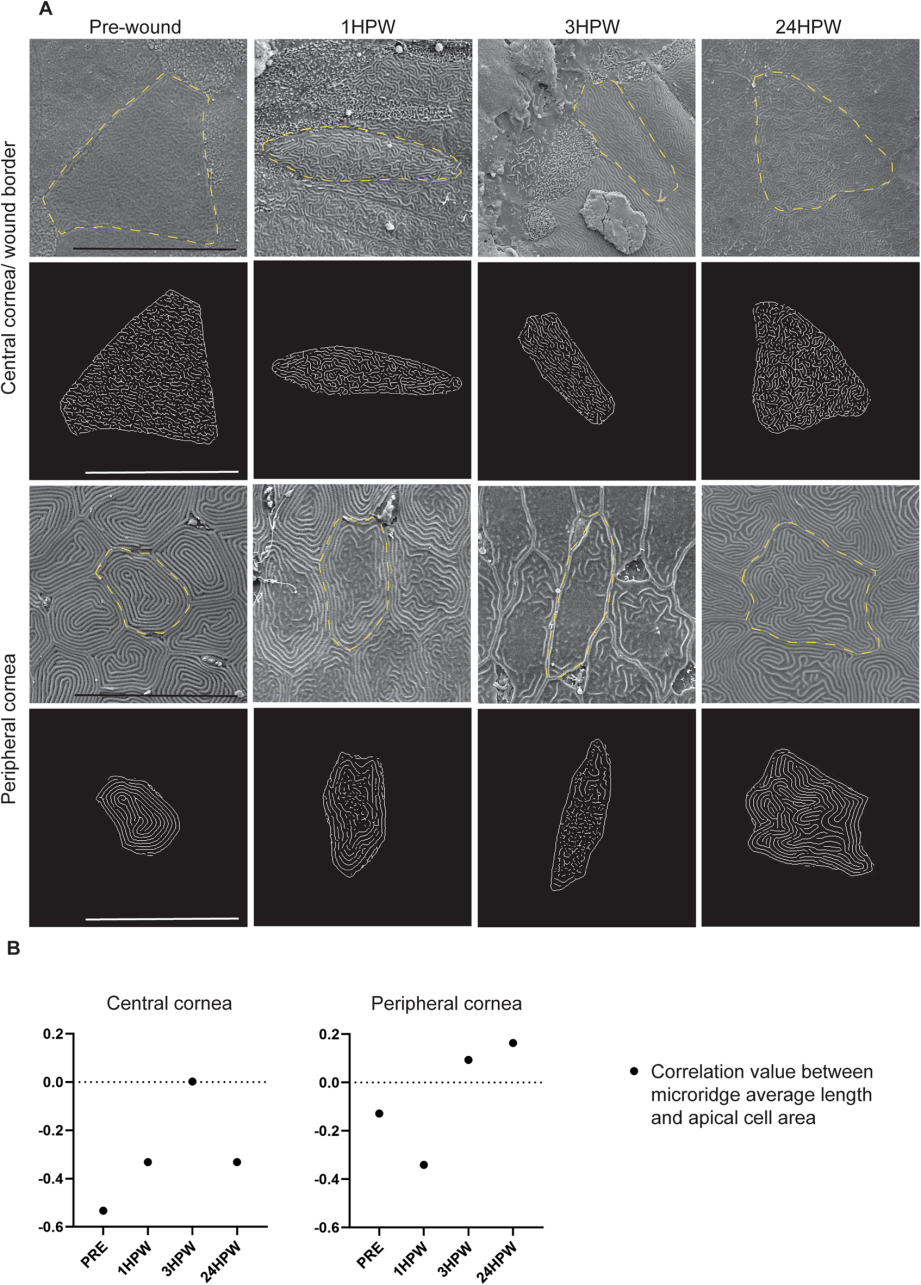
Microridge modification during corneal wound closing. (**A**) Representative SEM images and corresponding digitally generated microridge patterns before and 1, 3 and 24HPW on central and peripheral cornea. (**B**) Spearman correlation between average microridge length, and apical cell area, on central and peripheral cornea demonstrates the long-lasting modification of microridges during corneal wound healing (n-numbers after removal of outliers in pre-, 1, 3, and 24HPW, respectively: CC, microridge average length: 51, 47, 30, and 43, PC, microridge average length: 162, 171, 209, and 203). Data represent mean + 95% confidence interval, P** < 0.01, P*** < 0.001, P**** < 0.0001. B: Kruskal–Wallis test followed by Dunn’s test for multiple comparisons. Cells from 3 eyes were pooled for all analyses. CC: central cornea, PC: peripheral cornea. Scale bars: 20 µm. HPW: hours post-wound.

Consequently, we examined the changes which take place in microridge dynamics during wound closure (Fig. 7A), analyzing the correlation value of microridge average length and cell size. When negative, this correlation value reflects a higher cell area and a lower microridge average length, which indicates a high turn-over. When positive, this correlation value indicates that the microridges are more stable. Our correlation analysis demonstrated that during wound closure, central corneal cells displayed a progressive increase in microridge stability, while peripheral corneal cell showed a transitory destabilization of the microridges, followed rapidly by an increased stabilization (Fig. 7B).

### Corneal transcriptomic signature indicates a role for TGF-beta pathway during wound closure

**Figure 8:**
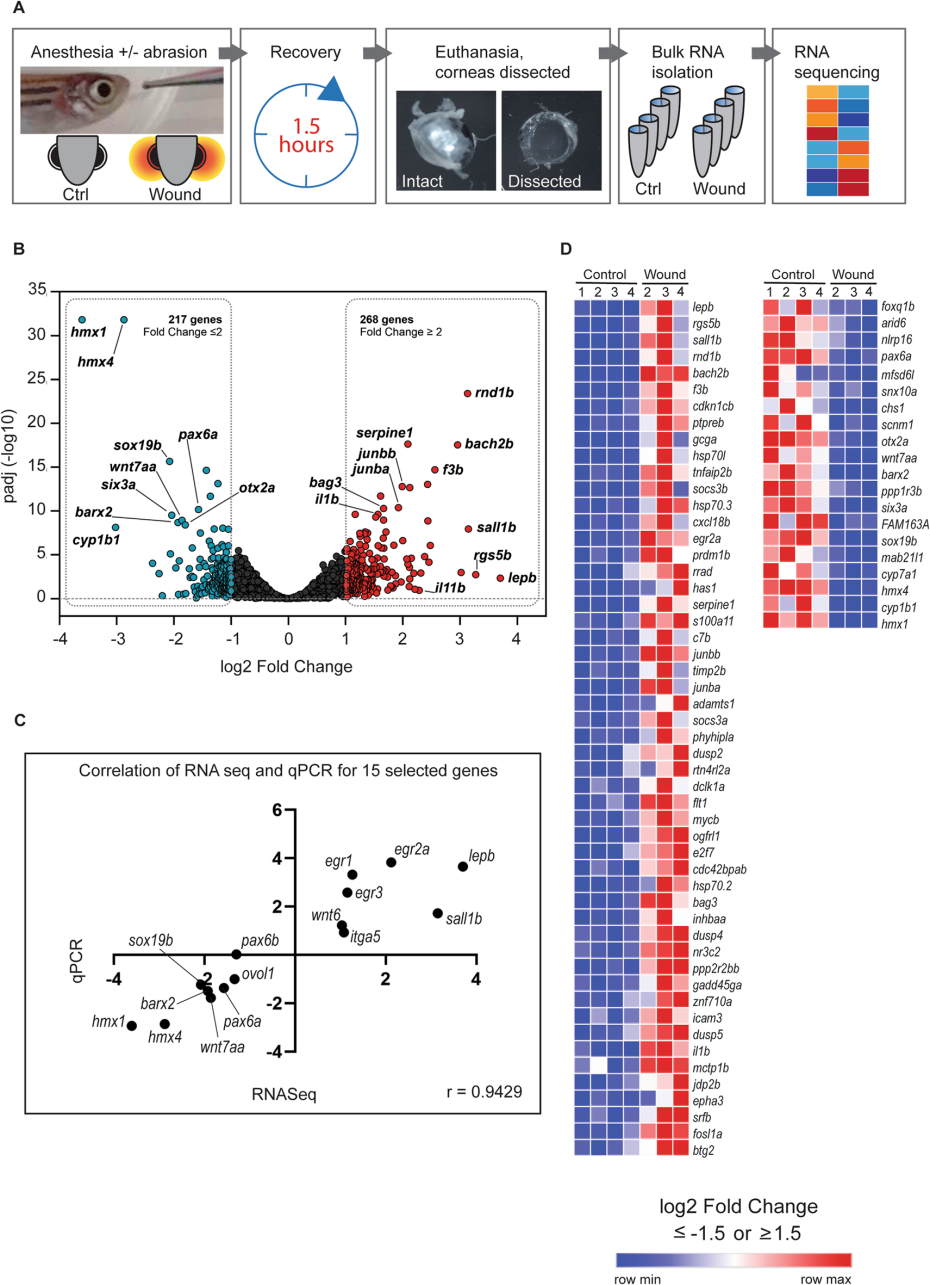
Transcriptomic changes during corneal wound closure. (**A**) Schematic presentation of the abrasion experiment, corneal collection and sample processing for RNA sequencing. (**B**) Volcano plot of gene expression changes (wounded vs. control samples) displays the up- and down-regulated genes at 1.5HPW. (**C**) Pearson correlation of RNA sequencing and qPCR results for selected 15 genes is used to validate the RNA-Sequencing results. (**D**) The heat map presents 52 most upregulated and 20 most downregulated genes (Log2 fold change ≥ 1.5 and ≤ − 1.5, p-adjusted ≤ 0.05). HPW: hours post-wound.

Because of the rapid induction of cell proliferation and shape modification, along with actin cytoskeleton turnover alteration, we investigated modulations of transcriptomic signature during corneal wound healing. We analyzed the zebrafish corneal transcriptomic signature during wound closure, at 1.5HPW (Fig. 8, Table S1). Comparison of control *versus* wounded corneas identified 268 genes being up- and 217 genes being down-regulated by a fold equal to or greater than 2 (Fold Change ≥ 2) (Fig. 8B). We further validated the results of 15 genes by qPCR (Fig. 8C). The correlations obtained using RNA sequencing and qPCR showed similar trends. Notably, a number of genes related to eye and visual system development were significantly downregulated in wounded corneas (Fig. 8D and Fig. S2). These include genes expressing the transcription factors *pax6a*, *hmx1*, *six3a* and *otx2a*, as well as *cyp1b1*—a gene important for zebrafish^29^ and for human ocular physiology^30^.

**Figure 9:**
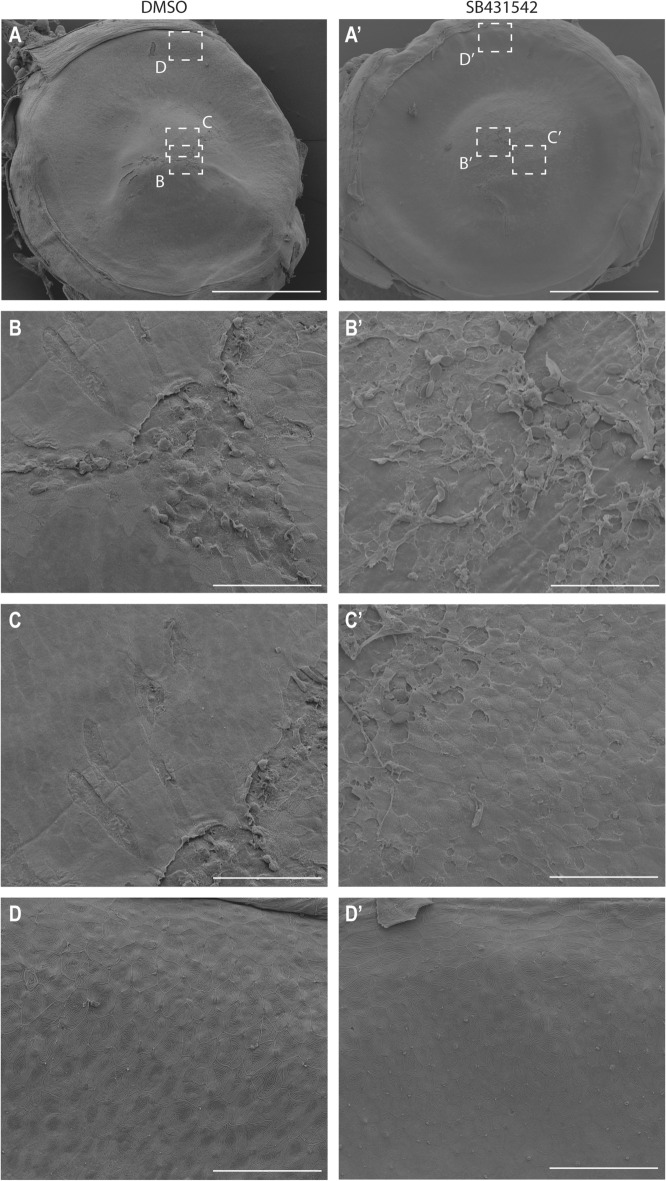
TGF-beta receptor inhibition delays wound closure. SEM images showing the wound site, a site adjacent to the wound site, and peripheral/limbal cornea at 3HPW in DMSO (**A**–**D**) versus TGF-beta receptor I inhibitor (SB431542, (**A’**–**D’**)) -treated fish. (**A**, **A’**) Overview of the eye, indicating the locations of areas in (**B**–**D’**). (**B**,**B’**) The wound site exhibits a greater disorganization under TGF-beta receptor inhibition. (**C**,**C’**). While the wound border has a smooth surface in the control, the TGF-beta receptor inhibition leads to a defected surface. (**D**,**D’**). In the peripheral region, the TGF-beta receptor inhibition seems to not affect drastically epithelial cells. Scale bars: 500 µm (**A**,**A’**), 50 µm (**B**,**B’**,**C**,**C’**,**D**,**D’**). HPW: hours post-wound.

**Figure 10:**
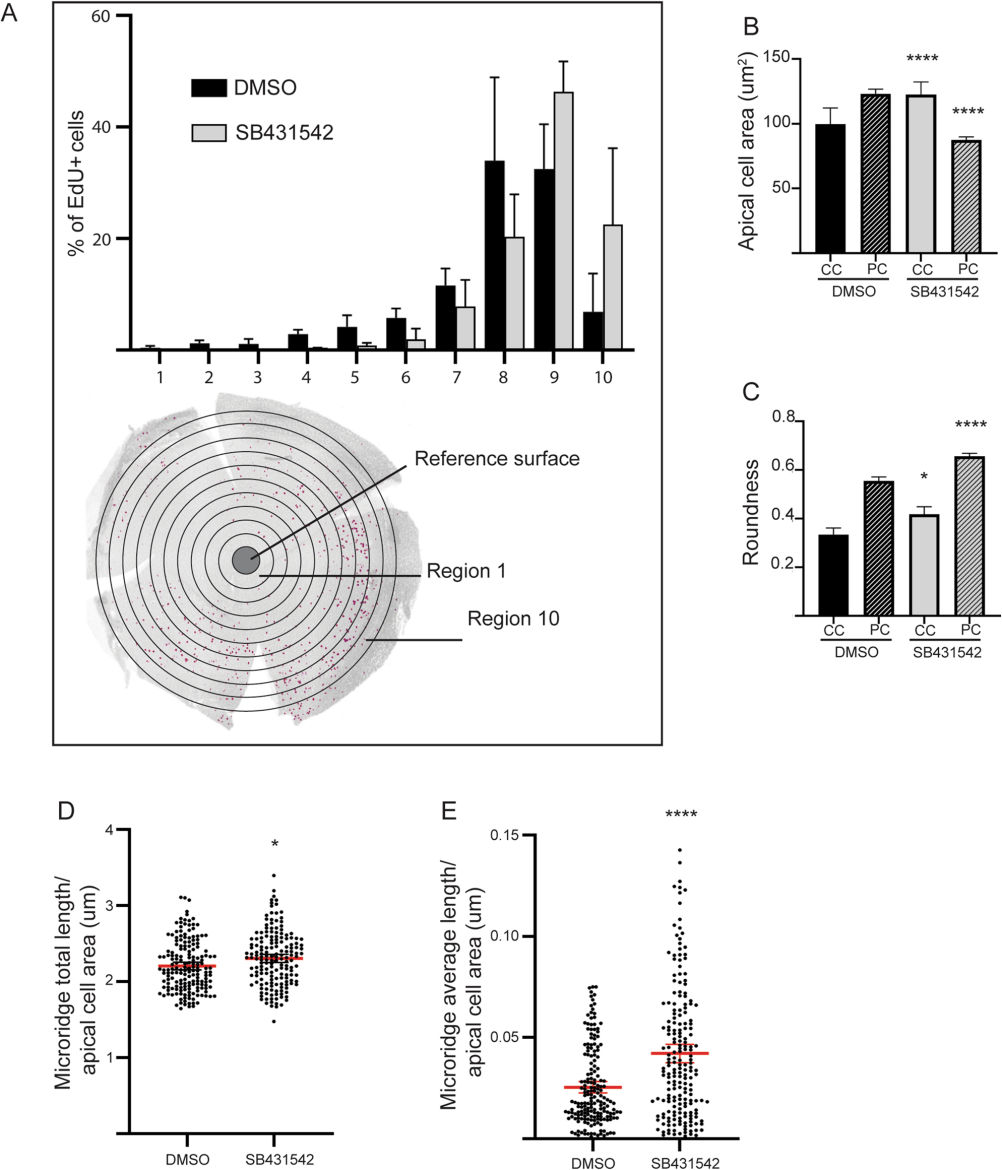
TGF-beta receptor inhibitor affects cell dynamics and morphology during wound closure. (**A**) The distribution of EdU+ cells, across the corneal radius from the central cornea (region 1) to the periphery (region 10), indicates a displacement of proliferation towards corneal periphery. n = 3 per treatment. (**B**) Quantification of apical cell area at 3HPW shows an increase of this parameter in central cornea and decrease in peripheral cornea upon TGF-beta receptor inhibition. Cells from 3 eyes were pooled for analysis (n-numbers after removal of outliers are: DMSO-CC, 88, DMSO-PC, 484, TGF-beta receptor inhibition-CC, 91, TGF-beta receptor inhibition-PC, 513). (**C**) Quantification of roundness at 3HPW indicates and increase of roundness in central and peripheral cornea upon TGF-beta receptor inhibition. Cells from 3 eyes were pooled for analysis (n-numbers after removal of outliers are: DMSO-CC, 85, DMSO-PC, 484, TGF-beta receptor inhibition-CC, 91, TGF-beta receptor inhibition-PC, 515). (**D**,**E**) TGF-beta receptor inhibition leads to an increase of microridge total length (**D**), and average length (**E**), per cell at 3HPW on peripheral cornea. Cells from 3 eyes were pooled for analysis (n-numbers after removal of outliers are: DMSO, 175, TGF-beta receptor inhibition, 169 (**D**), DMSO, 185, TGF-beta receptor inhibition, 198 (**E**). Data represent mean + stdev (**A**), or mean + 95% confidence interval (**B**–**E**), P* < 0.05, P**** < 0.0001. Kruskal–Wallis test followed by Dunn’s test for multiple comparisons. HPW: hours post-wound.

We noticed that a number of the modulated genes were linked to the Transforming Growth Factor beta (TGF-beta) pathway (Fig. 8D), such as *Leptin*^31^, *Otx2*^32^, *Egr2*^33^, *Sall1*^34^, *Cyp1*^35^, *Jun-B*^36^ and *Bach2*^37^. The TGFbeta isoforms 1, 2, and 3, as well as TGFbeta receptors, are expressed in human cornea^38,39^, and TGFbeta signaling is recognized as important regulator in corneal injury responses^8,40,41^. In vivo wound healing experiments have shown elevated TGFbeta^42,43^ and TGFbeta receptor^44^ levels in recovering epithelium. TGF receptor II ablation in corneal epithelium delayed wound closure^45^, as did TGFbeta 3 silencing^42^.Given the evidence of the pivotal role of TGFbeta on corneal re-epithelialization, we investigated the impact of TGF-beta receptor I inhibition on corneal wound closure by adding SB431542, an Alk inhibitor acting on TGF-beta receptor I, to the tank water 3 h prior to and 3 h after abrasion (Fig. 9). The results were remarkable: in such conditions, epithelial cells seemed disorganized and unable to migrate collectively (Fig. 9B’,C’). Furthermore, the close-up SEM image showed very few microridges on the cell surface, at the wound site. As the wound closure appeared defective, we investigated several parameters known to be involved in the process. Notably, the EdU+ cells seemed to be localized further away from the wounding site when TGF-beta receptor was inhibited (Fig. 10A). Cell shape descriptors, such as apical cell area (Fig. 10B) and roundness (Fig. 10C), were also significantly altered by TGF-beta signaling inhibition. Finally, microridge total and average lengths were increased in peripheral cornea (Fig. 10D–E). These elements demonstrated that TGF-beta signaling regulation is crucial during corneal epithelial wound closure.

### *Pax6* expression is lost during corneal wound closure

**Figure 11:**
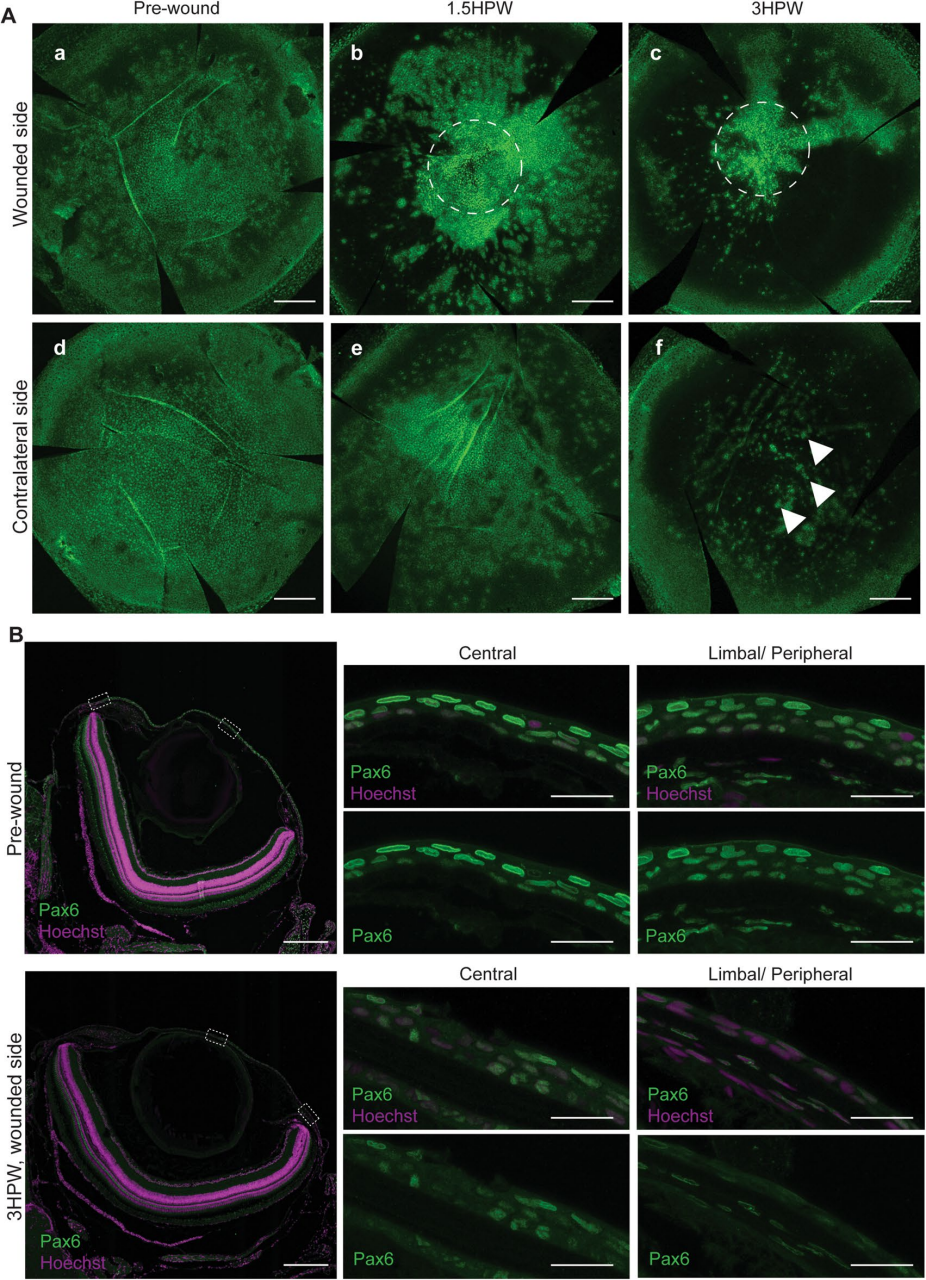
PAX6 immunological detection during wound closure. (**A**) Whole-mount staining showing an overview of Pax6 signal before wounding, and 1.5 or 3HPW, shows the disappearance of Pax6 in areas distant to the wound and a reinforcement closer to the wound site, on the wounded side (a–c). Dashed lines indicate wound location. On the contralateral side (d–f), Pax6 remains only in specific site, leading to a punctated pattern. (**B**) Immunofluorescence on sections showing Pax6 signal together with Hoechst nuclear counterstaining, before or 3HPW (wounded side) in central or peripheral cornea (boxed areas in whole-eye images), confirms the global loss of Pax6. HPW: hours post-wound. Maximum intensity projection of the whole cornea or 5-µm section, scale bars: 300 µm in A, 200 and 20 µm in B. HPW: hours post-wound.

Our transcriptomic analysis (Fig. 8B) identified another factor regulated by TGF-beta signaling, and essential for eye formation, namely pax6^46^. The zebrafish genome houses two *Pax6* genes, among which only *Pax6a* plays a role in the ocular tissues, while *Pax6b* is mainly found in the pancreas^47^. Pax6 transcription factor is known to be the master gene of eye formation (for review^48^). Previous studies have reported that in the context of *Pax6* mutation, corneal cells are unable to differentiate properly, and adopt the corneal epithelial cell identity^49^. After P*ax6a* expression was validated by qPCR in control and wounded corneas (Fig. 8C), we used immunohistochemistry to analyze Pax6 pattern during corneal wound healing. Before abrasion, Pax6 was found in all areas of the corneal epithelium (Fig. 11Aa, B). At 1.5HPW, we detected high levels of Pax6 immunoreactivity around the wound, while the rest of corneal epithelium was devoid of any Pax6 signal (Fig. 11Ab). By 3HPW, only a few cells, located near the wound site were Pax6 positive, while most of the cornea displayed no Pax6 signal (Fig. 11Ac, B). It was notable that a strong decrease of the Pax6 signal occured progressively during corneal wound healing in the contralateral eye (Fig. 11Ad–f), to the point where only a few cells became Pax6 positive by 3HPW (Fig. 11Af, arrows), thus indicating unequivocally a bilateral response to unilateral corneal abrasion. These observations were confirmed with immunostaining on paraffin sections (Fig. 11B).

### Bilateral response prepares a rapid epithelial wound healing

**Figure 12:**
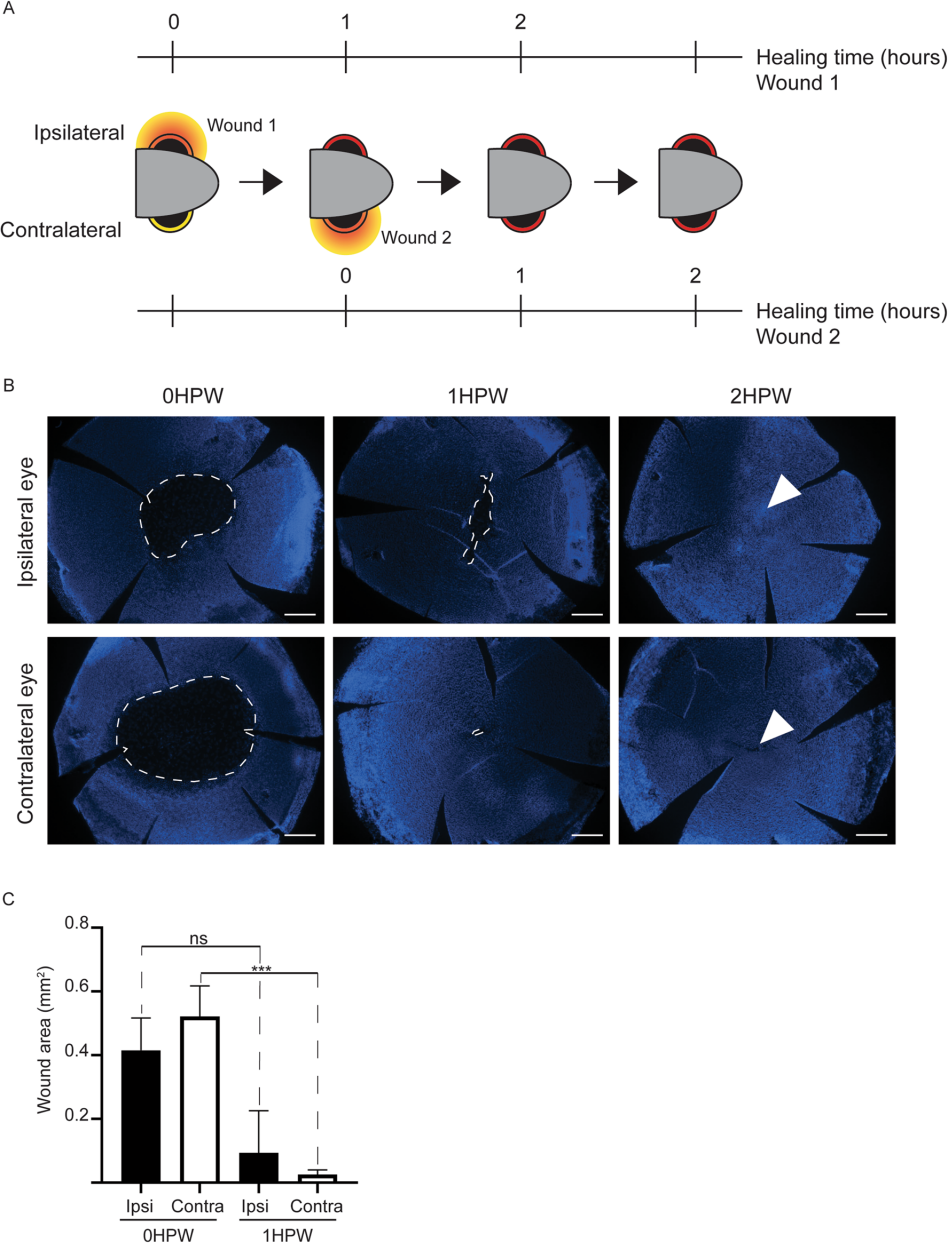
Accelerated wound closure on the contralateral cornea after abrasion. (**A**) A schematic presentation of the experiment. The second wound, on the contralateral side is performed 1HPW. Each wound closure is followed during 2 h. (**B**) Representative images of the cornea of either the ipsilateral or contralateral eye at 0, 1, and 2HPW (Hoechst-staining). Dashed line indicates wound border, arrowhead points to wound site in a closed wound. The wound on the contralateral side is closed by 1 HPW, while the wound is still largely open on the ipsilateral cornea at 1HPW. (**C**) Quantification of the wound area on the right and on the left side at 1 or 2HP confirms the faster closure on the contralateral eye. Data represent mean + stdev, P*** < 0.001, n = 5–6 per group. Kruskal–Wallis test followed by Dunn’s test for multiple comparisons. HPW: hours post-wound. Scale bars: 200 µm.

These strong cellular and molecular responses from an unharmed epithelium were unexpected. We therefore hypothesized that a bilateralization of corneal injury response might be beneficial in the case of a secondary injury to the contralateral eye, which would continue to provide a proper vision. We chose to inflict an abrasion on the contralateral eye, 1 h after abrading the ipsilateral eye, and then monitored the wound closure speed for 2 h after each wound (Fig. 12A). Strikingly, the wound closure efficacy was significantly higher on the contralateral eye (Fig. 12B–C). While 22% of the wound remained open at 1HPW on the ipsilateral cornea, after the same delay, there remained less than 5% of the wound still to be closed on the contralateral eye.

Collectively, our results confirm the bilateralization of corneal wound healing response, but more importantly point towards communication between both eyes.

## Discussion

While the zebrafish corneal structure is similar to corneas found in terrestrial animals equipped with camera-type eyes, it has never previously been used to delineate the corneal wound healing mechanism. Our study took advantage of this model to demonstrate that cell rearrangement is a key element during corneal wound closure. Furthermore, we have shown that the modification of the epithelial cell molecular signature, together with the modulation of specific pathways, such as TGF-beta, is important during wound closure. Taken together, these elements prove the crucial role played by cell plasticity during corneal wound closure.

Cell proliferation is often regarded as a key element in wound closure. That said, we and others have previously reported that corneal wound closure occurs in a mater of days in mice^9,17,22^ and in rabbits^50,51^, despite the fact that in mice, this phenomenon has been reported as being independent of cell proliferation induction^9^. Interestingly, our results from EdU labeling show that, unlike in mice, in zebrafish cell proliferation is induced quickly after wounding. We hypothesize that this difference between species could be attributed to one of the three following factors. First, corneal wound healing in mice is a lengthy process. Therefore, the lack of reported proliferation could reflect a short proliferation window which might not have been identified by previous studies. Second, corneal wound closure might not follow the same steps in mice and in zebrafish, due to innovations brought about by evolution of the visual apparatus. Third, the pool of available cells for wound closure is more restricted in zebrafish than in mice, requiring the production of more cells to close the wound. By blocking cell proliferation, however, we have disproved this last option. Furthermore, despite cell proliferation induction, our results are showing a fairly similar wound closing mechanism in zebrafish and in mice. Further investigation is therefore needed to decipher thoroughly the early steps of murine corneal closure, in order to identify the possible discrepencies between the mammalian and fish models, especially regarding cell migration, crucial element during epithelial wound healing.

Cell shape and apical microridge dynamics are fitted readouts for epithelial physiology. We demonstrated that cell shape modification was correlated with microridge dynamic modulation. While the microridge pattern in central corneal cells seemed to return to normal rapidly, peripheral corneal cells displayed a long term perturbation of microridges. Our observations indicate that the peripheral cornea is the driving force in wound closure, while wound border cells adopt a phenotype facilitating the epithelial cell displacement until closure is completed, unlike peripheral corneal cells, which demonstrate a longer phenotype disturbance. By synchronizing the differential response of these two regions, wound closure occurs more rapidly. This coordination must be under the control of specific molecular signals which harmonize corneal epithelial cell behaviour during wound closure.

Among the molecular changes our transcriptomic analysis revealed, during wound closure there appears to be heavy modulation of numerous factors involved in the TGF-beta pathway, and *Pax*6. Interestingly, a previous report demonstrated the role of TGF-beta signalling in the balance between corneal epithelial cell stemness and terminal differentiation, via the regulation of *Pax6*, among other factors^52^. This transcription factor is a known master gene for ocular structure identity, especially the cornea. When *Pax6* expression is absent during eye formation in a chick embryo, corneal identity fails to set in epithelial cells, and is replaced with skin phenotype^53^. Furthermore, a recent study demonstrated that *Pax6* is a crucial player for direct cell reprogramming into corneal epithelium^54^. We can conclude that *Pax6* level is crucial when enforcing corneal epithelium identity. The clear *Pax6* downregulation which we report here is most likely the reflection of an increase in cell plasticity, via a looser corneal identity maintenance. It is also of note that a recent report showed that terminally differentiated corneal cells can replace lost stem cells^19^. Our original finding, together with these studies, demonstrate that upon injury cell identity might be lessened to allow a rapid phenotype modification for wound closure. These observations can be viewed within the larger perspective of cell plasticity in organ regeneration. By attaining a higher degree of plasticity, terminally differentiated cells appear to develop the capacities to heal a tissue or an organ, as it has been observed in relation to skin^55^, the intestine^56^, and the vascular system^57^, among others. It is notable, however, that *Pax6* modulation has not been studied in the early stages of wound closure in mouse models, although an increase of *Pax6* expression later on in the healing process was reported^58^. It would therefore be of great interest to compare the cellular and molecular aspects of corneal wound closure and healing in various species to understand the conservation level of this molecular regulation.

Further investigations could shed new light on the molecular modulations of cell identity and phenotype required during wound healing and tissue regeneration, not only in cornea, but in most organs. It might then be possible, by selectively modifying the cell microenvironment, to induce sufficient plasticity in order to harness the regenerative capabilities.

One of the most interesting findings of our study is the bilateralization of corneal response to an unilateral injury. We ourselves have previously reported the bilateralization of lacrimal gland response in mice after abrasion^10^. Other studies on murine and human cornea have also described such a phenomenon^59,60^. Interestingly, the synchronization of both sides appears to be of importance, given that as it has been conserved during evolution. Moreover, very potent factors would be required to modify cell identity and the dynamics of a differentiated epithelial tissue in the absence of injury. Our results revealed changes to cell morphology, increased proliferation, and a modified identity. These results, therefore, raise three fundamental questions. First, what is the nature of the factors inducing cell plasticity and bilateralization? Second, what is the origin of these factors? Third, what advantage does bilateralization confer to an organism? While further investigations are necessary to find definite answers to these questions, the observations made in this study demonstrate that external cues can modify corneal epithelial identity, and that these cues can accelerate corneal would healing, a phenomenon which in the long run could be beneficial for patients suffering from corneal defects.

Collectively, our results demonstrate that a complex process is involved in early corneal wound healing. Besides the implication of cell rearrangements and morphological changes, wound closure also involves the induction of cell proliferation on the wound periphery, which appears to support global cell displacement towards the wound site. Moreover, profound transcriptomic changes are rapidly engaged subsequent to corneal insults. Taken together, these mechanisms are collectively responsible for rapid wound closure, and resemble the process observed in mammalian corneal wound healing. We are therefore confident that the use of zebrafish as a corneal regeneration model would reveal new aspects of corneal physiopathologies, which could be beneficial for the 28 million people worldwide suffering from uni- or bi-lateral corneal blindness.

## Methods

### Fish lines and fish maintenance

Fish were maintained in standard conditions with a 14 h:10 h light–dark cycle at the Zebrafish unit (HiLIFE, University of Helsinki). During maintenance and wound healing experiments, fish were fed one to three times a day.

In this study we used the WT AB background (acquired from the Zebrafish facility, University of Helsinki). Fish were collected at the age of four to seven months. The fish were randomly selected for each experiment from the stock tank.

### Corneal wounding

The fish were anesthetized with 0.02% Tricaine (A5040 Sigma-Aldrich, St. Louis, Missouri, USA) and placed into an incision on a moist sponge, with the head protruding from the sponge surface. Corneal epithelium wounding was performed as described in^61^ using an ophthalmic burr (Algerbrush II, Alger Company, Texas, USA) with a 0.5 mm diameter rounded-tip and applying moderate pressure on the eye surface. After abrasion, the fish were transferred to tank water for recovery. Control animals were anesthetized and placed on a sponge for an equal duration to the wounded animals.

### EdU-labeling, hydroxyurea treatment, TGF-beta receptor I inhibition

To labelling proliferative cells, fish were transferred to 0.2 mM EdU (900584, Sigma-Aldrich) for 1.5 h before collecting samples. Three individuals were treated in a minimum of 120 ml of freshly prepared EdU solution (in system water).

In hydroxyurea treatment, fish were preincubated in 50 mM hydroxyurea (400046, Sigma-Aldrich) solution (in system water) for 3 h, anesthetized (0.02% MS-222), and subjected to corneal abrasion, then kept in the hydroxyurea solution for 3 h before sample collection. For TGF-beta receptor I inhibition, fish were preincubated with 50 µM SB431542 (S4317, Sigma-Aldrich) solution (in system water) for 3 h, anesthetized (0.02% MS-222), and subjected to corneal abrasion, then kept in the inhibitor solution for 3 h before sample collection.

### Whole mount staining

Fish were euthanized at selected time points by anesthesia in 0.02% Tricaine solution followed by decapitation. For whole mount staining, whole heads or enucleated eyes were fixed for 20 min on ice in 4% PFA prepared in PBS from 20% stock solution (15713, Electron Microscopy Sciences, Hatfield, Pennsylvania, USA). The samples were rinsed with PBS and stored in 100% methanol at − 20 °C. The samples were rehydrated by incubation at RT in 75% methanol/25% PBS, 50% methanol/50% PBS, and 25% methanol/75% PBS, for 5 to 10 min each. For samples collected as whole heads, eyes were enucleated after this step in PBS. Tissue was permeabilized with 0.3% Triton-x-100/PBS, with rocking agitation 4 times for 5 min at RT, and blocked at RT several hours (10% normal goat serum (16210064, Life Technologies, Penrose, Auckland, New Zealand), 0.5% BSA (A2153, Sigma), in 0.1% Triton-x-100/PBS) with rocking agitation. Samples were incubated with a primary antibody (rabbit polyclonal antibody to Pax6, ab5790, Abcam, Cambridge, UK) 1:200 in blocking solution overnight at 4 °C with rocking agitation. Next, samples were washed in 0.1% Triton-x-100/PBS (fast rinse, 5 times for 5 min, then 3 times for 20 min), and blocked in blocking solution for 1 to 2 h at RT with rocking agitation. Samples were then incubated in secondary antibody (goat polyclonal antibody to rabbit IgG, A11008, Life Technologies) 1:200 in blocking solution, containing Hoechst 1:2000 for 2 to 3 h at RT with rocking agitation. Finally, samples were washed as above and stored in PBS at 4 °C until dissection. The cornea was dissected from the eye in PBS using fine scissors and tweezers and placed on a microscopy slide in a drop of 80% glycerol, and covered with a coverslip.

EdU-labeled tissue was stained for EdU with a kit (C10337, Invitrogen). When done in combination with antibody staining, EdU tracing followed the secondary antibody incubation step (Hoechst was excluded from the secondary antibody solution). Samples were rinsed in PBS and incubated in an EdU reaction cocktail for 60 min at RT. Samples were then washed in PBS, counterstained with Hoechst (1:2000 in PBS) for 20 min, washed in PBS, and stored in PBS before dissection and mounting. When not incubated in combination with antibody staining, EdU-labeled samples were rehydrated and permeabilized as above, and blocked in 3% BSA/PBS for 30 min at RT. The EdU staining was done as above, followed by washing in 3% BSA/PBS for 15 min. The staining was then continued as above.

### Stainings on paraffin-embedded, formalin-fixed sections

For histology, eye/ whole head samples were fixed overnight at 4 °C in 4% PFA. Then, samples were rinsed in PBS, mounted in Histogel (if eyes were processed), dehydrated in an increasing ethanol series, and embedded in paraffin. 5-µm sections were prepared with microtome, dried overnight at 37 °C, and attached to slides by brief baking at 65–70 °C. The sections were stored at 4 °C until use. Stainings on sections began with deparaffinization in Xylene and rehydration in a decreasing ethanol series. For Hematoxylin–eosin staining, slides were incubated in hematoxylin for 2 min, rinsed in tap water, incubated in eosin for 45–60 s, dehydrated in an increasing ethanol series and xylene, and embedded in a mounting medium.

For immunofluorescence, the sections were prepared and rehydrated as above, then subjected to permeabilization in 0.3% Triton-x-100/PBS for 10 min at RT, followed by heat-induced antigen retrieval in 10 mM sodium-citrate buffer, pH 6, in a 2100 Retriever machine (Aptum Biologics, Southampton, U.K.). Then, sections were washed 10 minutes in PBS, blocked for 60 min in 10% goat serum in 0.1% Triton-x-100/PBS, and incubated overnight in the primary antibody (rabbit polyclonal antibody to Pax6, ab5790, Abcam) 1:200 at RT in blocking solution. Next morning, samples were washed in 0.1% Triton-x-100/PBS for 15 min at RT, and incubated in the secondary antibody (goat polyclonal antibody to rabbit IgG, A11008, Life Technologies) 1:400 in blocking solution, containing Hoechst 1:2000 (H3570, Invitrogen) 2 h at RT. Finally, samples were washed in 0.1% Triton-x-100/PBS, and in PBS, then mounted with Fluoromount-G (0100–01, Southern Biotech, Birmingham, Alabama, USA).

For EdU detection on sections, after rehydration the samples were washed in 3% BSA/PBS for 6 min, permeabilized in 0.5% Triton-x-100/PBS for 20 min, incubated in the EdU reaction cocktail for 30 min, then washed in 3% BSA/PBS, and in PBS. All steps were done at RT.

### Image acquisition

Whole mount, and immunofluorescence on sections, of WT samples were imaged with the Leica SP8 inverted confocal microscope (Leica Microsystems, Wetzlar, Germany); sequential scanning was performed with HC PL APO 10x/0.40 CS2, HC PL APO 20x/0.75 CS2, or HC PL APO 63x/1.20 CS2 objective. For Pax6 or EdU, and Hoechst staining, laser lines and emission detection ranges were 405/430–480 (Hoechst) and 488/500–550 (Pax6/EdU). The images in Fig. 9B were taken with a Zeiss Axio Imager.M2 upright microscope, using EC Plan-Neofluoar objectives (5X/0.16, 10X/0.30). The Hoechst signal was detected with excitation at 335–383 nm, and emission at 420–470 nm. All images compared within each experiment were taken with similar settings.

### EdU signal quantification

For EdU quantification, tile scan images with 20X objective were acquired and merged in LAS software (Leica). The amount of EdU-positive cells was determined using Imaris 9.3 (Bitplane). EdU+ cells were selected with the ‘dots’ function and exported to Excel. For EdU+ cell localization, a column-shaped object extending from the top to the bottom of the image stack was placed in the center of the cornea, and the distance intensity of each EdU+ cell to the object was measured in Imaris. The raw values were exported to Excel, and normalized to the length of the corneal radius. The corneal radius was then divided into ten subregions, and the proportion of EdU+ cells in each region was calculated and presented as EdU+ cell percentage per region.

### Scanning electron microscopy

Fish were euthanized at selected time points by anesthesia in 0.02% Tricaine solution (Sigma-Aldrich) followed by decapitation. Whole heads were fixed overnight at + 4 °C in 2.5% glutaraldehyde (Grade 1, Sigma) in 0.1 M sodium-phosphate buffer pH 7.4. On the following day, samples were rinsed in 0.1 M sodium-phosphate buffer pH 7.4 three times. Eyes were dissected and stored in 0.1 M sodium-phosphate buffer pH 7.4 at + 4 °C until further processing at the Electron Microscopy unit (University of Helsinki). Samples were treated with osmium tetroxide (2%), dehydrated in increasing ethanol series and by critical point drying. Finally, the samples were coated with platinum. Images were taken with an FEI Quanta Field Emission Gun scanning electron microscope.

### Cell shape descriptor and microridge measurements

Scanning electron microscopy images were used for quantifications. Central cornea and 3–4 areas from peripheral cornea were imaged with 2000X magnification. Cell roundness and the apical area were measured using Fiji ImageJ 1.53^62^. A grid of crosses was superimposed onto the image, and both cells marked by crosses and those showing clear borders were selected with the polygon selection tool. Shape descriptors and area were measured for each cell. 3 eyes per condition were used for measurements. For the microridge total and the average length per cell, a subset of cells selected for shape descriptor analysis was analysed as follows. The cell was selected with the polygon selection tool, and the background was cleared. The image was then smoothened 1–3 times, brightness and contrast were adjusted automatically, and the image was convolved and converted into a binary image. Finally, the pattern was skeletonized, and the ‘analyze skeleton’ function was used to measure microridge values.

### RNA-sequencing and computational analysis

For RNA-sequencing, same-age fish were grouped one day prior to the experiment into four tanks (14 fish per 3 L). Aged-matched (145, 148, 180 or 183 day-old) control and wound specimens were collected from the same tank. With respect to wounding, abrasion in both corneas of the animals was performed as described above. Control animals were anesthetized and placed onto a sponge for an equal duration as for the wounded animals. After 1.5 h recovery, the fish were anesthetized again in 0.02% Tricaine and decapitated. Eyes were enucleated and cornea dissected in PBS. For one sample, 6 corneas from 3 individuals were pooled in 100 µl of Tri reagent (T9424, Sigma). RNA was isolated from TRI reagent (T9424, Sigma) using Precellys beads and standard protocols (P000912-LYSK0-A.0, Bertin Technologies, Montigny-Le-Bretonneux, France). RNA was further purified using the Qiagen Rneasy MiniElute Cleanup kit (#74204, Qiagen, Hilden, Germany). Polyadenylated mRNA was purified from 50 ng of Total RNA using NEBNext Poly(A) mRNA Magnetic Isolation Module (#E7490, New England Biolabs, Ipswich, Massachusetts, USA).

The following steps were performed by the Biomedicum Functional Genomics Unit (FuGu) at the Helsinki Institute of Life Science and Biocenter Finland at the University of Helsinki. Library preparation was completed on purified mRNA using the NEBNext Ultra Directional RNA Library prep kit ( #E7420L, New England Biolabs) using 14 cycles of PCR amplification and indexed using single (i7) indexing. Indexed library preps from each sample were then pooled and sequenced at a pool concentration of 1.3 pM on the NextSeq 500 using a NextSeq High Output 75 cycle flow cell (Illumina, San Diego, California, USA) with 75SE reads. Basecalling and demultiplexing was performed using Illumina bcl2fastq (v2.20.0.422) software. Reads were mapped to zebrafish GRCz11 genome using STAR aligner (2.6.0c). Gene counts were calculated using the featureCounts tool from the Subread package (v1.22.2) using Ensembl release 97 zebrafish gtf-files. Differential expression analysis was performed using R package DESeq2 (v1.22.2^63^). One sample was excluded based on visual PCA inspection (Fig. S2A). For downstream analyses, only genes with an entrezgene id were consider, therefore excluding predicted genes (Table S1). Gene Ontology (GO) term analysis was performed using g:Profiler (version e98_eg45_p14_ce5b097^64^) online tool with default parameters. Volcano plots and heatmaps were generated using GraphPad Prism 8 (version 8.3.0, Graphpad Software, San Diego, California, USA).

### Statistical analysis

Data were analyzed in Graphpad Prism 8 software. Data was tested for normality, and due to at least one non-normally distributed group in all data sets, non-parametric tests were used. For experiments with small n-number, non-parametric test was chosen by default. Differences between two groups were tested with Man-Whitney two-tailed test. Differences between multiple groups were tested with Kruskal–Wallis test, followed by Dunn’s multiple comparisons test. Correlations were studied with Spearman’s correlation.

### Ethical approval

All aspects of the animal works were approved by the Finnish National Board of Animal Experimentation (ESAVI/22167/2018). All methods were carried out in accordance with relevant guidelines and regulations and followed the ARRIVE guidelines.

### Illustrations

All figure panels were designed and produced with Adobe Illustrator 2021, including the schematic drawing of the Fig. 1.

## Supplementary Information

Supplementary Information 1.

Supplementary Information 2.

## References

[1] Ayala FJ (2007). Darwin's greatest discovery: Design without designer. PNAS.

[2] Zieske JD (2004). Corneal development associated with eyelid opening. Int. J. Dev. Biol..

[3] Klenkler B, Sheardown H, Jones L (2007). Growth factors in the tear film: Role in tissue maintenance, wound healing, and ocular pathology. Ocul. Surf..

[4] Guier, C. P. & Stokkermans, T. J. Cornea foreign body removal in *StatPearls.* NBK554478 (StatPearls, 2021). 32119365

[5] Moshirfar, M., Bennett, P. & Ronquillo, Y. Corneal dystrophy in *StatPearls*. NBK557865 (Statpearls, 2021). 32491788

[6] Dua HS, Gomes JA, Singh A (1994). Corneal epithelial wound healing. Br J Ophthalmol..

[7] Suzuki K (2003). Cell-matrix and cell–cell interactions during corneal epithelial wound healing. Prog. Retin. Eye Res..

[8] Ljubimov AV, Saghizadeh M (2015). Progress in corneal wound healing. Prog. Retin. Eye Res..

[9] Kalha S (2018). Bmi1+ progenitor cell dynamics in murine cornea during homeostasis and wound healing. Stem Cells.

[10] Kuony A (2019). Ectodysplasin-A signaling is a key integrator in the lacrimal gland-cornea feedback loop. Development.

[11] Parravicini R (2012). Tuna cornea as biomaterial for cardiac applications. Tex. Heart Inst. J..

[12] Dhouailly D, Pearton DJ, Michon F (2014). The vertebrate corneal epithelium: From early specification to constant renewal. Dev. Dyn..

[13] Lehrer MS, Sun TT, Lavker RM (1998). Strategies of epithelial repair: modulation of stem cell and transit amplifying cell proliferation. J. Cell Sci..

[14] Fabiani C, Barabino S, Rashid S, Dana MR (2009). Corneal epithelial proliferation and thickness in a mouse model of dry eye. Exp. Eye Res..

[15] Strinkovsky L, Havkin E, Shalom-Feuerstein R, Savir Y (2021). Spatial correlations constrain cellular lifespan and pattern formation in corneal epithelium homeostasis. Elife.

[16] Park M (2019). Peripheral (not central) corneal epithelia contribute to the closure of an annular debridement injury. PNAS.

[17] Park M (2019). Visualizing the contribution of keratin-14(+) limbal epithelial precursors in corneal wound healing. Stem Cell Rep..

[18] Chang CY, Green CR, McGhee CN, Sherwin T (2008). Acute wound healing in the human central corneal epithelium appears to be independent of limbal stem cell influence. Invest. Ophthal. Vis. Sci..

[19] Nasser W (2018). Corneal-committed cells restore the stem cell pool and tissue boundary following injury. Cell Rep..

[20] Altshuler A (2021). Discrete limbal epithelial stem cell populations mediate corneal homeostasis and wound healing. Cell Stem Cell.

[21] Farrelly O (2021). Two-photon live imaging of single corneal stem cells reveals compartmentalized organization of the limbal niche. Cell Stem Cell.

[22] Li Y (2021). The common YAP activation mediates corneal epithelial regeneration and repair with different-sized wounds. NPJ Regen. Med..

[23] Pan YA (2013). Zebrabow: Multispectral cell labeling for cell tracing and lineage analysis in zebrafish. Development.

[24] Salic A, Mitchison TJ (2008). A chemical method for fast and sensitive detection of DNA synthesis in vivo. PNAS.

[25] Richardson R (2016). Re-epithelialization of cutaneous wounds in adult zebrafish combines mechanisms of wound closure in embryonic and adult mammals. Development.

[26] Pinto CS (2019). Microridges are apical epithelial projections formed of F-actin networks that organize the glycan layer. Sci. Rep..

[27] Lam PY, Mangos S, Green JM, Reiser J, Huttenlocher A (2015). In vivo imaging and characterization of actin microridges. PLoS ONE.

[28] Collin HB, Collin SP (2000). The corneal surface of aquatic vertebrates: microstructures with optical and nutritional function?. Philos. Trans R. Soc. Lond. B Biol. Sci..

[29] Williams AL, Eason J, Chawla B, Bohnsack BL (2017). Cyp1b1 regulates ocular fissure closure through a retinoic acid-independent pathway. Invest. Ophthalmol. Vis. Sci..

[30] Li N, Zhou Y, Du L, Wei M, Chen X (2011). Overview of cytochrome P450 1B1 gene mutations in patients with primary congenital glaucoma. Exp. Eye Res..

[31] Yan C, Yang Q, Shen HM, Spitsbergen JM, Gong Z (2017). Chronically high level of tgfb1a induction causes both hepatocellular carcinoma and cholangiocarcinoma via a dominant Erk pathway in zebrafish. Oncotarget.

[32] Jia S, Wu D, Xing C, Meng A (2009). Smad2/3 activities are required for induction and patterning of the neuroectoderm in zebrafish. Dev. Biol..

[33] Gregory KJ, Morin SM, Schneider SS (2017). Regulation of early growth response 2 expression by secreted frizzled related protein 1. BMC Cancer.

[34] Lin Y (2021). SALL1 regulates commitment of odontoblast lineages by interacting with RUNX2 to remodel open chromatin regions. Stem Cells.

[35] Muller GF, Dohr O, El-Bahay C, Kahl R, Abel J (2000). Effect of transforming growth factor-beta1 on cytochrome P450 expression: Inhibition of CYP1 mRNA and protein expression in primary rat hepatocytes. Arch. Toxicol..

[36] Klein K, Habiger C, Iftner T, Stubenrauch F (2020). A TGF-beta- and p63-responsive enhancer regulates IFN-kappa expression in human keratinocytes. J. Immunol..

[37] Kim EH (2014). Bach2 regulates homeostasis of Foxp3+ regulatory T cells and protects against fatal lung disease in mice. J. Immunol..

[38] Joyce NC, Zieske JD (1997). Transforming growth factor-beta receptor expression in human cornea. Invest. Ophthalmol. Vis. Sci..

[39] Nishida K (1994). Immunohistochemical localization of transforming growth factor-beta 1, -beta 2, and -beta 3 latency-associated peptide in human cornea. Invest. Ophthalmol. Vis. Sci..

[40] Yu FS, Yin J, Xu K, Huang J (2010). Growth factors and corneal epithelial wound healing. Brain Res. Bull..

[41] Wilson SE (2020). Corneal wound healing. Exp Eye Res..

[42] Bettahi I (2014). Genome-wide transcriptional analysis of differentially expressed genes in diabetic, healing corneal epithelial cells: hyperglycemia-suppressed TGFbeta3 expression contributes to the delay of epithelial wound healing in diabetic corneas. Diabetes.

[43] Huh MI, Chang Y, Jung JC (2009). Temporal and spatial distribution of TGF-beta isoforms and signaling intermediates in corneal regenerative wound repair. Histol. Histopathol..

[44] Zieske JD, Hutcheon AE, Guo X, Chung EH, Joyce NC (2001). TGF-beta receptor types I and II are differentially expressed during corneal epithelial wound repair. Invest. Ophthalmol. Vis. Sci..

[45] Terai K (2011). Crosstalk between TGF-beta and MAPK signaling during corneal wound healing. Invest. Ophthalmol. Vis. Sci..

[46] Jin M, Gao D, Wang R, Sik A, Liu K (2020). Possible involvement of TGFbetaSMADmediated epithelialmesenchymal transition in prometastatic property of PAX6. Oncol. Rep..

[47] Kleinjan DA (2008). Subfunctionalization of duplicated zebrafish pax6 genes by cis-regulatory divergence. PLoS Genet..

[48] Shaham O, Menuchin Y, Farhy C, Ashery-Padan R (2012). Pax6: A multi-level regulator of ocular development. Prog. Retin. Eye Res..

[49] Roux LN (2018). Modeling of aniridia-related keratopathy by CRISPR/Cas9 genome editing of human limbal epithelial cells and rescue by recombinant PAX6 protein. Stem cells..

[50] Pfister RR (1975). The healing of corneal epithelial abrasions in the rabbit: a scanning electron microscope study. Invest. Ophthal..

[51] Crosson CE, Klyce SD, Beuerman RW (1986). Epithelial wound closure in the rabbit cornea. A biphasic process. Invest. Ophthalmol. Vis. Sci..

[52] Hu L (2019). Expansion and maintenance of primary corneal epithelial stem/progenitor cells by inhibition of TGFbeta receptor I-mediated signaling. Exp. Eye Res..

[53] Collomb E (2013). The corneal epithelium and lens develop independently from a common pool of precursors. Dev. Dyn..

[54] Kitazawa K (2019). Direct reprogramming into corneal epithelial cells using a transcriptional network comprising PAX6, OVOL2, and KLF4. Cornea.

[55] Tang H (2020). MicroRNA-200b/c-3p regulate epithelial plasticity and inhibit cutaneous wound healing by modulating TGF-beta-mediated RAC1 signaling. Cell Death Dis..

[56] Kurokawa K, Hayakawa Y, Koike K (2020). Plasticity of intestinal epithelium: Stem cell niches and regulatory signals. Int. J. Mol. Sci..

[57] Tombor LS (2021). Single cell sequencing reveals endothelial plasticity with transient mesenchymal activation after myocardial infarction. Nat. Commun..

[58] Sivak JM (2000). Pax-6 expression and activity are induced in the reepithelializing cornea and control activity of the transcriptional promoter for matrix metalloproteinase gelatinase B. Dev Biol..

[59] Cruzat A (2015). Contralateral clinically unaffected eyes of patients with unilateral infectious keratitis demonstrate a sympathetic immune response. Invest. Ophthalmol. Vis. Sci..

[60] Sagga N, Kuffova L, Vargesson N, Erskine L, Collinson JM (2018). Limbal epithelial stem cell activity and corneal epithelial cell cycle parameters in adult and aging mice. Stem Cell Res..

[61] Kalha S, Kuony A, Michon F (2018). Corneal epithelial abrasion with ocular burr as a model for cornea wound healing. J. Vis. Exp..

[62] Schindelin J (2012). Fiji: an open-source platform for biological-image analysis. Nat Methods..

[63] Love MI, Huber W, Anders S (2014). Moderated estimation of fold change and dispersion for RNA-seq data with DESeq2. Genome Biol..

[64] Raudvere U (2019). g:Profiler: A web server for functional enrichment analysis and conversions of gene lists (2019 update). Nucleic Acids Res..

