# Supplementary material for: Unilateral zebrafish corneal injury induces bilateral cell plasticity supporting wound closure
Source: Sci Rep. 2022 Jan 7;12:161. doi: 10.1038/s41598-021-04086-x (PMC8741998; doi:10.1038/s41598-021-04086-x)
Supplement: Supplementary file 1 — Supplementary Information 1. [file 41598_2021_4086_MOESM1_ESM.pdf]

Unilateral Zebrafish corneal injury induces bilateral cell plasticity supporting wound closure

Supplementary material

Supplementary Figure S1. Wounded area stained with Hematoxylin-Eosin shows the progressive wound closure.
Scale bars: 20 µm. HPW: hours post-wound.

Supplementary Figure S2. Further analysis of RNA-Sequencing experiment. A. Principal component analysis of showing all samples initially collected, and the sample (wound #1) excluded from the final gene expression analysis. **B.** Gene ontology analysis (biological process) for genes with the log2 fold-change ≥1.5 or ≤ -1.5 (p-adjusted ≤0.05).

Supplementary Table S1. RNA-Sequencing analysis of corneal transcriptomic signature during corneal wound healing

Figure S1

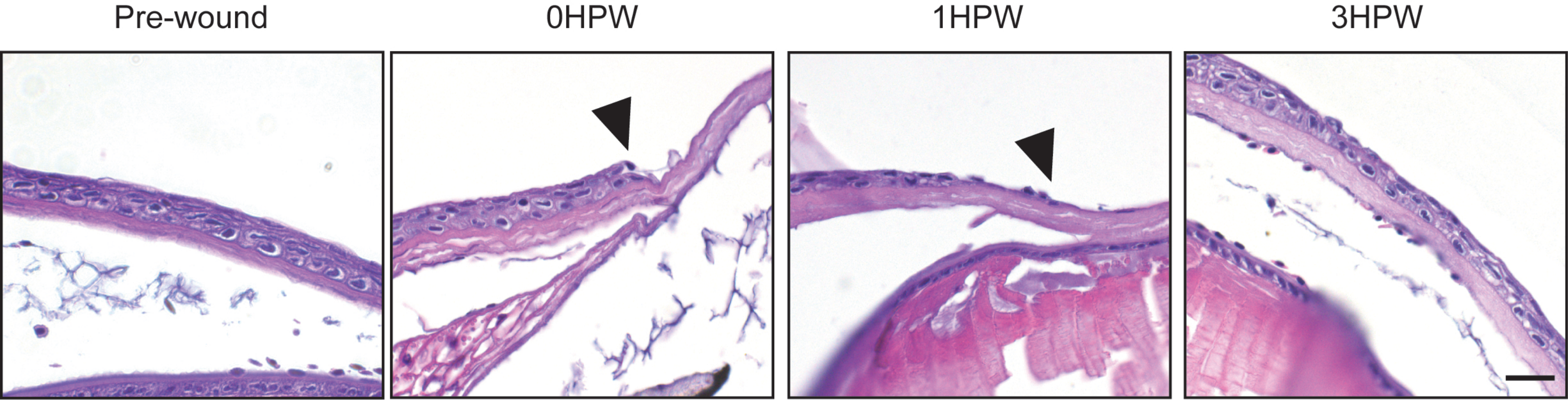

Figure S2

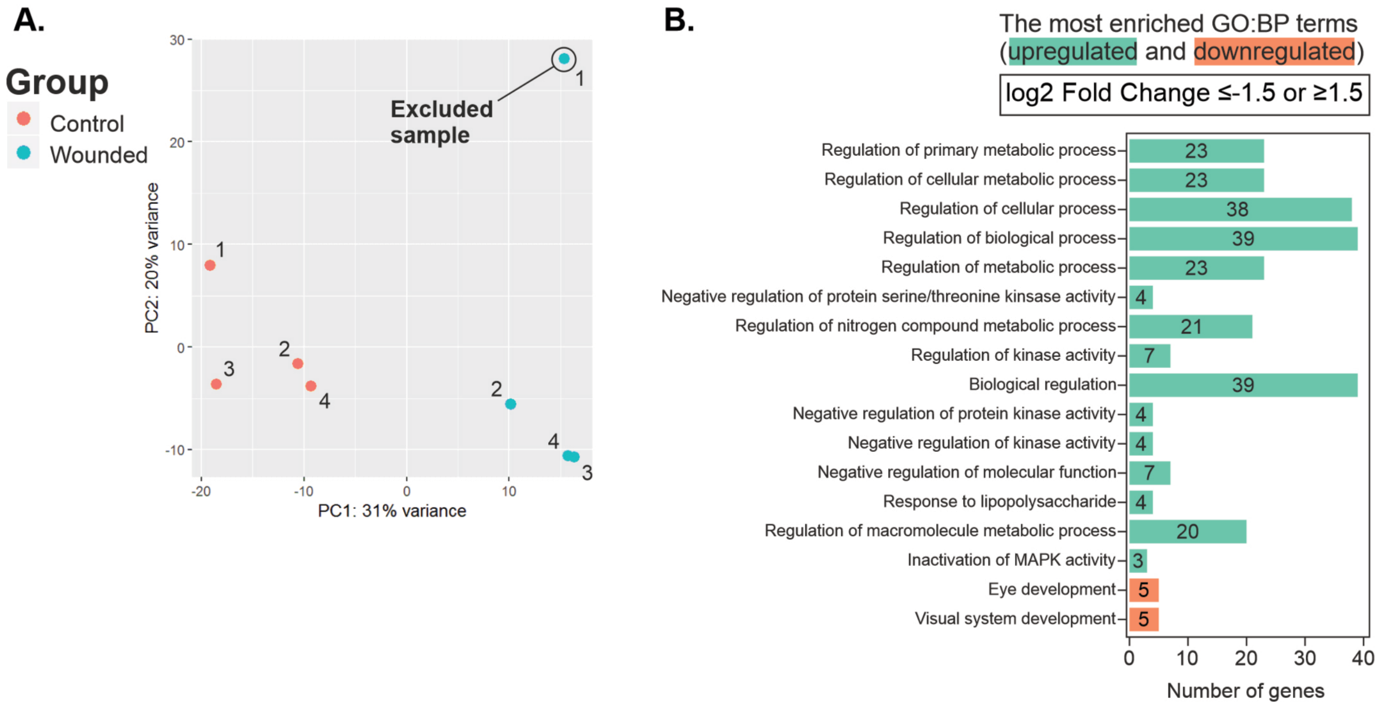
A.
Group
Control
Wounded
Excluded sample
PC2: 20% variance
PC1: 31% variance
B.
The most enriched GO:BP terms
(upregulated and downregulated)
log2 Fold Change ≤-1.5 or ≥1.5
Regulation of primary metabolic process
Regulation of cellular metabolic process
Regulation of cellular process
Regulation of biological process
Regulation of metabolic process
Negative regulation of protein serine/threonine kinsase activity
Regulation of nitrogen compound metabolic process
Regulation of kinase activity
Biological regulation
Negative regulation of protein kinase activity
Negative regulation of kinase activity
Negative regulation of molecular function
Response to lipopolysaccharide
Regulation of macromolecule metabolic process
Inactivation of MAPK activity
Eye development
Visual system development
Number of genes
